# Free fatty acids and mortality among adults in the United States: a report from US National Health and Nutrition Examination Survey (NHANES)

**DOI:** 10.1186/s12986-024-00844-6

**Published:** 2024-09-10

**Authors:** Meng Li, Lijing Zhang, Bi Huang, Yang Liu, Yang Chen, Gregory Y. H. Lip

**Affiliations:** 1 Department of Cardiology, Dongzhimen Hospital, Beijing University of Chinese Medicine, Beijing, China; 2 Liverpool Centre for Cardiovascular Science at University of Liverpool, Liverpool John Moores University and Liverpool Heart and Chest Hospital, William Henry Duncan Building, 6 West Derby Street, Liverpool, L7 8TX UK; 3 Department of Cardiology, The First Affiliated Hospital of Chongqing Medical University, Chongqing, China; 4 Department of Cardiovascular Medicine, The Second Affiliated Hospital, Jiangxi Medical College, Nanchang University, Nanchang, Jiangxi China; 5 Danish Center for Health Services Research, Department of Clinical Medicine, Aalborg University, Aalborg, Denmark

**Keywords:** Free fatty acids, All-cause mortality, Cardiovascular mortality, NHANES

## Abstract

**Background:**

The relationship between free fatty acids (FFAs) and the risk of mortality remains unclear. There is a scarcity of prospective studies examining the associations between specific FFAs, rather than total concentrations, of their effect on long-term health outcomes.

**Objective:**

To evaluate the correlation between different FFAs and all-cause and cardiovascular mortality in a large, diverse, nationally representative sample of adults in the US, and examine how different FFAs may mediate this association.

**Methods:**

This cohort study included unsaturated fatty acids (USFA) and saturated fatty acids (SFA) groups in the US National Health and Nutrition Examination Survey (NHANES) from 2011 to 2014 and provided blood samples for FFAs levels. Multiple model calibration was performed using Cox regression analysis for known risk factors to explore the associations between FFAs and all-cause and cardiovascular mortality.

**Results:**

In the group of USFA, 3719 people were included, median follow-up, 6.7 years (5.8–7.8 years). In the SFA group, we included 3900 people with a median follow-up, 6.9 years (5.9-8 years). In the USFA group, myristoleic acid (14:1 n-5) (hazard ratio (HR) 1.02 [1.006–1.034]; *P* = 0.004), palmitoleic acid (16:1 n-7) (HR 1.001 [1.001–1.002]; *P* < 0.001), cis-vaccenic acid (18:1 n-7) (HR 1.006 [1.003–1.009]; *P* < 0.001), nervonic acid (24:1 n-9) (HR 1.007 [1.002–1.012]; *P* = 0.003), eicosatrienoic acid (20:3 n-9) (HR 1.027 [1.009–1.046]; *P* = 0.003), docosatetraenoic acid (22:4 n-6) (HR 1.024 [1.012–1.036]; *P* < 0.001), and docosapentaenoic acid (22:5 n-6) (HR 1.019 [1.006–1.032]; *P* = 0.005) were positively associated with the all-cause mortality, while docosahexaenoic acid (22:6 n-3) had a statistically lower risk of all-cause mortality (HR 0.998 [0.996–0.999]; *P* = 0.007). Among the SFA group, palmitic acid (16:0) demonstrated a higher risk of all-cause mortality (HR 1.00 [1.00–1.00]; *P* = 0.022), while tricosanoic acid (23:0) (HR 0.975 [0.959–0.991]; *P* = 0.002) and lignoceric acid (24:0) (HR 0.992 [0.984–0.999]; *P* = 0.036) were linked to a lower risk of all-cause mortality. Besides 23:0 and 24:0, the other FFAs mentioned above were linearly associated with the risks of all-cause mortality.

**Conclusions:**

In this nationally representative cohort of US adults, some different FFAs exhibited significant associations with risk of all-cause mortality. Achieving optimal concentrations of specific FFAs may lower this risk of all-cause mortality, but this benefit was not observed in regards to cardiovascular mortality.

**Supplementary Information:**

The online version contains supplementary material available at 10.1186/s12986-024-00844-6.

## Introduction

Fatty acids, structurally, can be categorized as saturated fatty acids (SFA) (no double bonds) and unsaturated fatty acids (USFA); the later are again of monounsaturated fatty acids (MUFA) (one double bond), and polyunsaturated fatty acids (PUFA) (greater than one double bond) [1]. Functionally, the regulation of fatty acid metabolism is well-established in healthy individuals [2], emphasizing the significance of both specific type and quantity of consumed fatty acid. For over 50 years, reducing intake of SFA was recommended [3] given that SFA increase low-density lipoprotein cholesterol [4], a strong risk factor for cardiovascular disease (CVD). However, USFA, particularly PUSFA, may play a vital role in disease prevention and contribute to overall health [1]. Considering that essential fatty acids are exclusively obtained from dietary sources, their concentrations in blood, also known as free fatty acids (FFAs), could serve as indicators of dietary intake [5].

Although large quantities of FFAs may be supplied by diet, they may also be indicators of disease risk, morbidity, and mortality [6]. Several studies have displayed an association between FFAs concentrations and coronary artery disease (CAD) [7, 8], and FFAs may independently predict all-cause mortality and cardiovascular mortality in individuals with angiographic CAD [9]. In contrast, findings suggest that FFAs concentrations may not be an absolute risk factor [8] and are not related to the presence of angiographic CAD [9]. The discrepancies in these results could stem from the diverse effects that each individual fatty acid rather than groups of fatty acids may exert on CVD risk factors [10]. A study revealed that only palmitic acid association with increased risk of cardiovascular mortality but not the other SFA or the sum of all SFA [10]. Studies have found that high levels of circulating very long SFA was associated with a lower risk of coronary heart disease (CHD) [11] and heart failure [12]. All these results indicated that each fatty acid individually had different association with CHD [13].

Furthermore, the quality, rather than the quantity, of dietary fatty acids is paramount in influencing the development of cardiometabolic diseases [14]. Besides CAD, FFAs have emerged as a major link among obesity, development of metabolic syndrome, and atherosclerosis [15–17]. Marked elevations of FFAs have been observed in obesity and type 2 diabetes [17–21], as well as in patients with stroke [22]. Nevertheless, additional data elucidate that FFAs are used as an independent risk factor for cancer mortality, but presenting no association with myocardial infarction or overall cardiovascular mortality [23, 24].

Therefore, the relationship between different FFAs and the risk of mortality remains unclear. In this study, we aimed a nationally representative cohort from the US to investigate the association of individual FFAs with all-cause and cardiovascular mortality in adults.

## Methods

### Study population

The National Health and Nutrition Examination Survey (NHANES) is a program that conducts health surveys representative of the national civilian, noninstitutionalized population across the US.

Administered by the National Center for Health Statistics (NCHS) within the Centers for Disease Control and Prevention (CDC), NHANES serves as a comprehensive effort to evaluate the health and nutrition status of US citizens. NHANES stands out due to its comprehensive approach, encompassing both questionnaire data collected through in-person interviews and health examinations conducted in the mobile examination center, collecting specimens for laboratory tests. Consequently, strata, sample weights, and primary sampling units were utilized in accordance with the NHANES analytic guidelines [25] to address oversampling of specific subpopulations, the unequal probability of selection, and adjustments for nonresponse. The NHANES protocol received approval from NCHS ethics review board, and written consent was obtained from all participants.

**Fig. 1:**
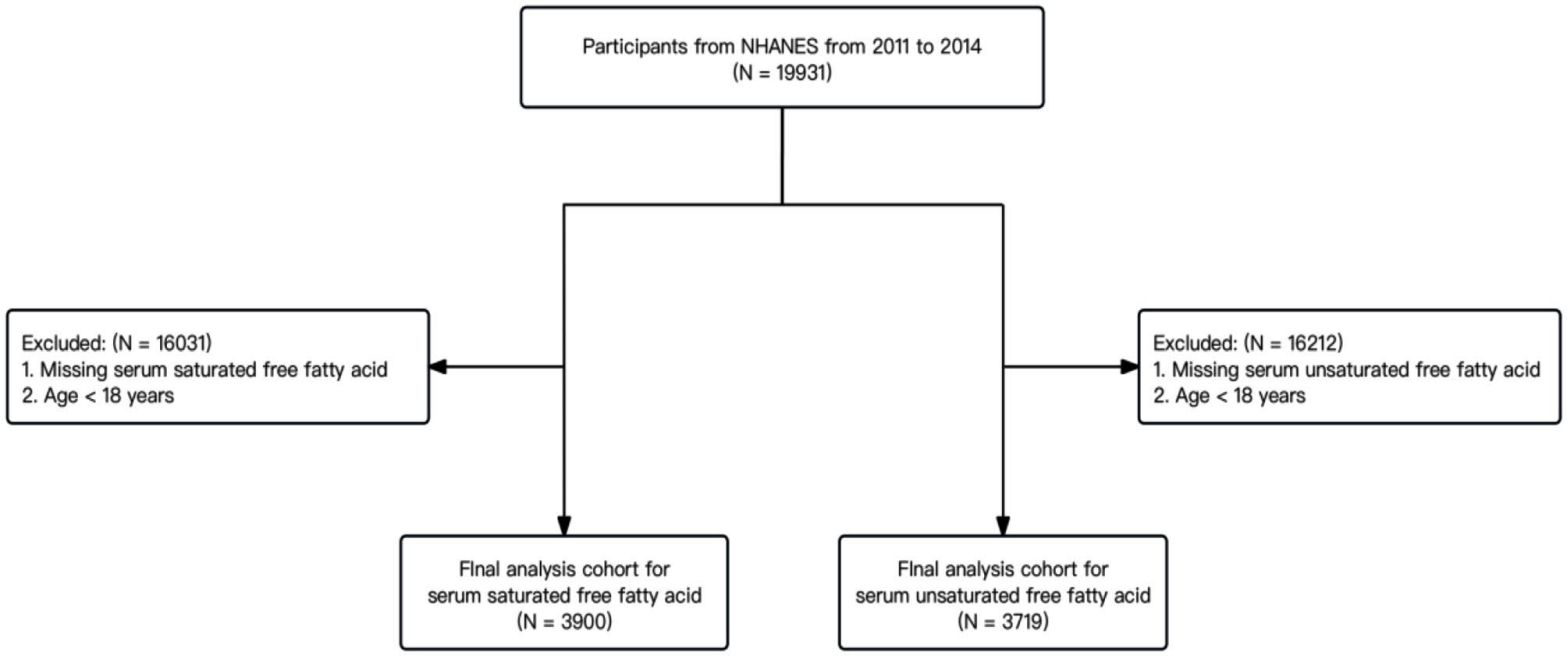
Flow chart for study. NHANES, National Health and Nutrition Examination Survey

For the present analysis, we included adults aged 18 and older who participated in NHANES during 2011–2014, which had available data on serum FFAs measurements. This study followed the guidelines of strengthening the reporting of observational studies in epidemiology for cohort studies. The proportions of missing values for the covariates we included were no more than 10%, so we used the “miceforest” in Python to implement multiple imputation by chained equations [26]. The final analysis was conducted on a cohort consisting of individuals who actively took part in the NHANES survey and had their each FFAs levels measured. The flow chart for this study showed in Fig. 1.

### Assessment of serum FFAs

Morning fasting blood samples were collected from NHANES participants, and serum FFAs were measured using a modified of Lagerstedt methods described previously [27]. Specifically, total fatty acids were hexane-extracted from the matrix, along with an internal standard solution containing eighteen stable isotopically-labeled fatty acids, to ensure accurate fatty acids recovery. The resulting extract was then derivatized to form pentafluorobenzyl esters and injected into a capillary gas chromatograph column to resolve individual fatty acids from other matrix constituents. FFAs were expressed as a % of total fatty acids (FFAs/the sum of measured fatty acids). Values of FFAs below the limit of detection divided by the square root of 2 [28]. A total of thirty fatty acids, including eleven SFA and nineteen USFA, were measured.

### Ascertainment of mortality outcomes

We utilized the NHANES public-use linked mortality file, linked by the NCHS to the national death index through December 31, 2019, employing a probabilistic matching algorithm to establish mortality status [29]. The US data on the underlying cause of death were utilized for case definition based on the international classification of diseases, 10th Revision [30], which death from heart disease (codes I00-I09, I11, I13, I20-I51, and I60-I69) are classified as cardiovascular mortality. This method has been confirmed by the CDC and has been utilized in previously CDC reports [31, 32].

### Assessment of covariates

The baseline information on age, sex, race, educational level, family income to poverty ratio (FIP), alcohol drinking, body mass index (BMI), abdominal obesity, weight loss, increasing exercise, cholesterol and triglycerides, and dietary intake (energy, protein, carbohydrate, sugar, dietary fiber, total fat, total SFA, total MUSFA and total PUSFA), comorbidities (diabetes, CVD, and cancer) were collected using questionnaires.

For all-cause and cardiovascular mortality, we adjusted for different combination of confunders, which were based on univariate logistic regression (Supplementary Table S1 for all-cause mortality, Supplementary Table S2 for cardiovascular mortality). We then utilized the variance inflation factor to address multicollinearity and removed the confunders with values exceeding 10 (Supplementary Table S3 for all-cause and cardiovascular mortality).

### Statistical analysis

Baseline characteristics and distribution of FFAs were compared between survivors and non-survivors. Continuous variables were expressed as median with interquartile range (for non-normal distribution), and the Mann-Whitney test was utilized for analysis. Categorical variables were displayed as counts (percentages) and underwent comparison through the chi-square test. FFAs associated with all-cause and cardiovascular mortality were based on univariate and multivariate Cox regression analysis. We utilized the Cox proportional hazards model to calculate hazard ratio (HR) and 95% confidence interval (CI). Quartile concentration (Q1, Q2, Q3, and Q4) of FFAs related to all-cause and cardiovascular mortality were found through Cox regression. Kaplan–Meier survival curves for all-cause mortality according to FFAs quartiles. To find the interactions with gender, we used subgroup analysis. We employed Restricted Cubic Spline (RCS) analysis to assess the non-linear association between FFAs and all-cause and cardiovascular mortality. Four knots were placed at the 25th, 50th, 75th, and 95th percentiles to define the spline. The follow-up duration for each individual was determined as the interval between the NHANES examination date and the last known date of being alive or being censored from the linked mortality file. We added a sensitivity analysis to further explore whether homeostatic model assessment for insulin resistance (HOMA-IR) would affect the results.

A *P*-value below 0.05 was considered statistically significant, and all analyses were two-tailed. Kaplan–Meier survival curves and RCS were generated using R software (version 4.3.2, Salzburg, Austria), while statistical analyses were performed with SPSS software (version 29, IBM).

## Results

### Baseline participant characteristics

As showed in Table 1, in the group of USFA, there were 796 (21.4%) adults aged ≥ 65 years and 1797 (48.3%) male. Between survivors and non-survivors, all social demographic (age, sex, race, education level and FIP) and comorbidities (diabetes, hypertension, chronic heart failure (CHF), angina, CHD, heart attack, stroke and cancer) were significantly different. All dietary habits showed statistical significance except for fat reduction and SFA intake. During 3719 person-years of follow-up (median follow-up, 6.7 years (5.8–7.8 years); maximum follow-up, 9.3 years), 321 deaths occurred, comorbid with 99 diabetes, 227 hypertension, 52 CHF, 57 CHD, 29 angina, 48 heart attack, 48 stroke, and 82 cancer.

**Table 1:** Baseline characteristic of serum unsaturated fatty acid and saturated fatty acid in the NHANES study, 2011–2014

Variables	Unsaturated fatty acid (*N* = 3719)				Saturated fatty acid (*N* = 3900)			
	All	Survivors	Non-survivors	*P*	All	Survivors	Non-survivors	*P*
Age ≥ 65 years	796 (21.4)	572 (16.8)	224 (69.8)	< 0.001	859 (22.0)	618 (17.4)	241 (70.3)	< 0.001
Male	1797 (48.3)	1605 (47.2)	192 (59.8)	< 0.001	1880 (48.2)	1678 (47.2)	202 (58.9)	< 0.001
Race				< 0.001				< 0.001
Mexican American	423 (11.4)	401 (11.8)	22 (6.9)		446 (11.4)	424 (11.9)	22 (6.4)	
Other Hispanic	407 (10.9)	391 (11.5)	16 (5.0)		396 (10.2)	381 (10.7)	15 (4.4)	
Non-Hispanic White	1599 (43.0)	1398 (41.1)	201 (62.6)		1584 (40.6)	1379 (38.8)	205 (59.8)	
Non-Hispanic Black	753 (20.2)	689 (20.3)	64 (19.9)		881 (22.6)	807 (22.7)	74 (21.6)	
Non-Hispanic Asian	451 (12.1)	436 (12.8)	15 (4.7)		506 (13.0)	486 (13.7)	20 (5.8)	
Other Race	86 (2.3)	83 (2.4)	3 (0.9)		87 (2.2)	80 (2.2)	7 (2.0)	
Education level				0.001				< 0.001
< High school	857 (23.0)	758 (22.3)	99 (30.8)		897 (23.0)	787 (22.1)	110 (32.1)	
High school	866 (23.3)	787 (23.2)	79 (24.6)		897 (23.0)	813 (22.9)	84 (24.5)	
College or higher	1996 (53.7)	1853 (54.5)	143 (44.5)		2106 (54.0)	1957 (55.0)	149 (43.4)	
FIP				< 0.001				< 0.001
< 0.5	295 (7.9)	278 (8.2)	17 (5.3)		307 (7.9)	287 (8.1)	20 (5.8)	
0.5-1	597 (16.1)	536 (15.8)	61 (19.0)		625 (16.0)	559 (15.7)	66 (19.2)	
1–2	962 (25.9)	850 (25.0)	112 (34.9)		1037 (26.6)	915 (25.7)	122 (35.6)	
> 2	1865 (50.1)	1734 (51.0)	131 (40.8)		1931 (49.5)	1796 (50.5)	135 (39.4)	
Alcohol drinking				0.646				0.724
Never	1071 (28.8)	975 (28.7)	96 (29.9)		1105 (28.3)	1005 (28.3)	100 (29.2)	
Ever	2648 (71.2)	2423 (71.3)	225 (70.1)		2795 (71.7)	2552 (71.7)	243 (70.8)	
BMI, kg/m^2^				0.306				0.356
< 25	1182 (31.8)	1069 (31.5)	113 (35.2)		1272 (32.6)	1152 (32.4)	120 (35.0)	
≥ 25 < 30	1197 (32.2)	1094 (32.2)	103 (32.1)		1242 (31.8)	1129 (31.7)	113 (32.9)	
≥ 30	1340 (36.0)	1235 (36.3)	105 (32.7)		1386 (35.5)	1276 (35.9)	110 (32.1)	
Abdominal obesity, cm				0.107				0.258
< 102 (Male) or < 88 (Female)	1712 (46.0)	1578 (46.4)	134 (41.7)		1819 (46.6)	1669 (46.9)	150 (43.7)	
≥ 102 (Male) or ≥ 88 (Female)	2007 (54.0)	1820 (53.6)	187 (58.3)		2081 (53.4)	1888 (53.1)	193 (56.3)	
Losing weight				0.256				0.156
Yes	2242 (60.3)	2058 (60.6)	184 (57.3)		2379 (61.0)	2182 (61.3)	197 (57.4)	
Increasing exercise				< 0.001				< 0.001
Yes	2211 (59.5)	2051 (60.4)	160 (49.8)		2322 (59.5)	2157 (60.6)	165 (48.1)	
Reducing salt diet				0.002				< 0.001
Yes	1909 (51.3)	1716 (50.5)	193 (60.1)		2025 (51.9)	1817 (51.1)	208 (60.6)	
Reducing fat diet				0.488				0.595
Yes	2038 (54.8)	1868 (55.0)	170 (53.0)		2134 (54.7)	1951 (54.8)	183 (53.4)	
Daily intake								
Energy, kcal	1954.0 (1450.0, 2582.0)	1970.5 (1465.0, 2598.0)	1754.0 (1301.5, 2382.5)	< 0.001	1956.0 (1446.0, 2573.0)	1971.0 (1458.5, 2595.5)	1755.0 (1318.0, 2369.0)	< 0.001
Protein, gm	75.4 (53.8, 102.5)	76.2 (54.6, 103.3)	68.1 (49.3, 93.6)	< 0.001	75.9 (53.8, 101.5)	76.6 (54.6, 102.8)	69.1 (49.0, 93.6)	< 0.001
Carbohydrate, gm	237.0 (170.3, 315.5)	239.9 (172.1, 320.0)	211.0 (155.9, 278.5)	< 0.001	235.5 (168.3, 313.3)	237.7 (170.0, 317.4)	211.0 (156.9, 279.1)	< 0.001
Sugar, gm	95.5 (59.0, 142.8)	96.5 (58.7, 144.1)	87.8 (59.5, 123.4)	0.018	94.7 (57.7, 140.8)	95.4 (57.4, 142.2)	88.9 (61.6, 121.6)	0.115
Dietary fiber, gm	14.9 (9.8, 21.9)	15.0 (9.9, 22.1)	13.9 (8.6, 19.9)	0.005	14.9 (9.7, 22.2)	15.1 (9.7, 22.5)	14.1 (9.2, 19.6)	0.015
Total fat, gm	72.5 (48.7, 102.3)	73.2 (49.1, 102.5)	64.9 (44.4, 96.4)	0.008	72.2 (48.7, 101.6)	72.7 (49.2, 102.4)	64.2 (44.2, 96.1)	0.007
Total SFA, gm	22.7 (14.7, 33.7)	22.8 (14.7, 33.9)	20.7 (13.9, 31.6)	0.074	22.3 (14.5, 33.6)	22.5 (14.6, 33.9)	20.7 (13.6, 31.8)	0.092
Total MUFA, gm	25.1 (16.6, 36.5)	25.3 (16.8, 36.7)	23.0 (15.0, 33.9)	0.020	25.4 (16.6, 36.5)	25.5 (16.9, 36.7)	22.6 (14.7, 34.2)	0.011
Total PUFA, gm	16. 2 (10.5, 24.8)	16.4 (10.7, 24.9)	14.2 (9.6, 23.4)	0.007	16.4 (10.4, 24.7)	16.5 (10.6, 24.8)	14.7(9.3, 23.0)	0.009
Laboratory indicators								
Total cholesterol, mg/dL	187.0 (161.0, 213.0)	187.0 (162.0, 214.0)	177.0 (153.0, 210.0)	< 0.001	186.0 (160.0, 213.8)	186.0 (161.0, 214.0)	178.0 (152.0, 213.0)	0.005
HDL-C, mg/dL	51.0 (43.0, 62.0)	51.0 (43.0, 62.0)	50.0 (42.0, 62.0)	0.185	52.0 (43.0, 62.0)	52.0 (43.0, 62.0)	51.0 (42.0, 63.0)	0.576
LDL-C, mg/dL	110.0 (87.0, 134.0)	111.0 (88.0, 135.0)	98.0 (76.0, 127.0)	< 0.001	10.08 (86.0, 133.0)	109.0 (87.0, 134.0)	98.0 (76.0, 129.0)	< 0.001
Triglycerides, mg/dL	97.0 (68.0, 146.0)	96.0 (68.0, 144.0)	106.0 (77.5, 160.0)	< 0.001	97.0 (68.0, 143.0)	95.0 (67.0, 141.0)	105.0 (77.0, 157.0)	< 0.001
Comorbidities								
Diabetes	432 (11.6)	333 (9.8)	99 (30.8)	< 0.001	459 (11.8)	355 (10.0)	104 (30.3)	< 0.001
Hypertension	1342 (36.1)	1115 (32.8)	227 (70.7)	< 0.001	1411 (36.2)	1165 (32.8)	246 (71.7)	< 0.001
CHF	142 (3.8)	90 (2.6)	52 (16.2)	< 0.001	145 (3.7)	91 (2.6)	54 (15.7)	< 0.001
CHD	147 (4.0)	90 (2.6)	57 (17.8)	< 0.001	156 (4.0)	95 (2.7)	61 (17.8)	< 0.001
Angina	102 (2.7)	73 (2.1)	29 (9.0)	< 0.001	107 (2.7)	75 (2.1)	32 (9.3)	< 0.001
Heart attack	145 (3.9)	97 (2.9)	48 (15.0)	< 0.001	156 (4.0)	105 (3.0)	51 (14.9)	< 0.001
Stroke	149 (4.0)	101 (3.0)	48 (15.0)	< 0.001	153 (3.9)	102 (2.9)	51 (14.9)	< 0.001
Cancer	339 (9.1)	257 (7.6)	82 (25.5)	< 0.001	357 (9.2)	269 (7.6)	88 (25.7)	< 0.001

“losing weight” and “increasing exercise” were assessed by the question “Are you now losing weight” and “Are you now increasing exercise”, respectively, collected by medical conditions recall interviews, “reducing salt diet, reducing fat diet” were assessed by the question “Have you done Dietary information (reducing salt diet and reducing fat diet)” collected by 24-hour dietary recall interviews, from which total energy intake was calculated using the US Department of Agriculture Automated Multiple-Pass Method; the Alternative Healthy Eating Index (AHEI) is based on a comprehensive review of the original Healthy Eating Index and subsequent studies that included food components (energy, protein, carbohydrate, sugar, dietary fiber, total fat, total SFA, total MUFA and total PUFA. Abbreviations: FIP, family income to poverty ratio; BMI, body mass index, SFA, saturated fatty acids; MUFA, monounsaturated fatty acids; PUFA, polyunsaturated fatty acids; HDL-C, high-density lipoprotein cholesterol; LDL-C, low-density lipoprotein cholesterol; CHF, congestive heart failure; CHD, coronary heart disease

In the SFA group, we included 859 (22.0%) adults aged ≥ 65 and 1880 (48.2%) males. Baseline characteristics in the SFA population were consistent between survivors and non-survivors, except for sugar intake. During 3900 person-years of follow-up (median follow-up, 6.9 years (5.9-8 years), maximum follow-up, 9.3 years), 343 deaths occurred, comorbid with 104 diabetes, 246 hypertension, 54 CHF, 61 CHD, 32 angina, 51 heart attack, 51 stroke, and 88 cancer (Table 1).

### Characteristics of FFAs

A total of 30 different FFAs were included in our study, including 19 USFA and 11 SFA. Table 2 showed that in the USFA group, a statistically significant difference was observed between survivors and non-survivors (palmitoleic acid (16:1 n-7), cis-vaccenic acid (18:1 n-7), Oleic acid (18:1 n-9), eicosenoic acid (20:1 n-9), linoleic acid (18:2 n-6), stearidonic acid (18:4 n-3), eicosatrienoic acid (20:3 n-9), arachidonic acid (20:4 n-6), docosatetraenoic acid (22:4 n-6), and docosapentaenoic acid (22:5 n-3)). In the SFA group, 6 fatty acids were statistically significant between the groups (capric acid (10:0), palmitic acid (16:0), margaric acid (17:0), docosanoic acid (22:0), tricosanoic acid (23:0), and lignoceric acid (24:0)). As shown in Table 2, non-survivors demonstrated higher concentrations of the USFA, 16:1 n-7, 18:1 n-7, 18:1 n-9, 20:1 n-9, 20:3 n-9, 20:4 n-6, 22:4 n-6, 22:5 n-3, docosapentaenoic acid (22:5 n-6), and docosahexaenoic acid (22:6 n-3). Among SFA, 10:0, 16:0, and 17:0 showed increased concentrations in the non-survivor group, while 22:0, 23:0 and 24:0 were lower compared to survivors.

**Table 2:** Distribution of serum fatty acids in the two cohorts

Name	All	Survivors	Non-survivors	*P*
Unsaturated fatty acids (µmol/L)				
Myristoleic acid (14:1 n-5)	6.0 (3.3, 10.0)	6.0 (3.3, 10.0)	6.0 (3.3, 10.0)	0.745
Palmitoleic acid (16:1 n-7)	199.0 (135.0, 298.0)	197.0 (133.0, 295.3)	228.0 (151.5, 320.0)	0.002
cis-Vaccenic acid (18:1 n-7)	141.0 (11.05, 177.0)	140.0 (114.0, 175.0)	156.0 (126.5, 194.0)	< 0.001
Oleic acid (18:1 n-9)	1930.0 (1550.0, 2500.0)	1920.0 (1540, 2480)	2110.0 (168.0, 2670.0)	< 0.001
Eicosenoic acid (20:1 n-9)	13.0 (10.1, 16.9)	13.0 (10.0, 16.6)	14.0 (11.0, 18.1)	< 0.001
Nervonic acid (24:1 n-9)	86.3 (73.0, 102.0)	86.0 (73.0, 102.0)	87.2 (73.0, 104.0)	0.578
Linoleic acid (18:2 n-6)	3480.0 (2960.0, 4070.0)	3500.0 (2970.0, 4080.0)	3340.0 (2735.0, 3985.0)	0.002
α-Linolenic acid (18:3 n-3)	77.0 (56.6, 107.0)	77.0 (57.0, 106.3)	76.2 (54.9, 111.0)	0.701
γ-Linolenic acid (18:3 n-6)	51.0 (35.8, 73.0)	51.0 (35.4, 73.0)	53.0 (38.9, 76.9)	0.085
Stearidonic acid (18:4 n-3)	3.0 (2.0, 5.0)	3.0 (2.0, 5.0)	3.0 (2.0, 5.0)	0.035
Eicosadienoic acid (20:2 n-6)	21.7 (17.6, 27.2)	21.6 (17.5, 27.0)	22.0 (18.0, 28.9)	0.121
Dihomo-γ-Linolenic acid (20:3 n-6)	151.0 (117.0, 194.0)	151.0 (117.0, 194.0)	147.0 (112.5, 190.0)	0.232
Eicosatrienoic acid (20:3 n-9)	7.0 (5.0, 9.4)	6.8 (4.9, 9.1)	8.0 (5.2,8.0)	< 0.001
Arachidonic acid (20:4 n-6)	823.0 (673.0, 1010.0)	820.0 (673.0, 1000.0)	864.0 (712.0, 1050.0)	0.004
Eicosapentaenoic acid (20:5 n-3)	50.7 (33.9, 79.0)	50.6 (33.1, 78.8)	52.5 (36.6, 82.8)	0.100
Docosatetraenoic acid (22:4 n-6)	25.3 (20.0, 32.5)	25.0 (20.0, 32.1)	27.8 (21.5, 34.4)	< 0.001
Docosapentaenoic acid (22:5 n-3)	48.7 (38.6, 62.0)	48.2 (38.2, 61.0)	52.0 (41.5, 65.6)	< 0.001
Docosapentaenoic acid (22:5 n-6)	19.7 (15.0, 25.9)	19.5 (14.9, 25.6)	20.2 (15.0, 26.7)	0.059
Docosahexaenoic acid (22:6 n-3)	144.0 (109.0, 199.0)	143.0 (109.0, 198.3)	149.0 (107.5, 199.5)	0.537
Saturated fatty acids (µmol/L)				
Capric acid (10:0)	1.7 (1.1, 2.9)	1.7 (1.1, 2.8)	1.9 (1.1, 3.0)	0.009
Lauric acid (12:0)	7.3 (4.8, 13.0)	7.3 (4.8, 12.8)	7.9 (5.0, 14.7)	0.295
Myristic acid (14:0)	105.0 (72.0, 157.0)	105.0 (72.0, 157.0)	104.0 (73.7, 153.0)	0.790
Pentadecanoic acid (15:0)	21.0 (16.0, 27.7)	21.0 (16.0, 27.5)	22.0 (16.0, 28.3)	0.358
Palmitic acid (16:0)	2630.0 (2160.0, 3230.0)	2620.0 (2160.0, 3220.0)	2730.0 (2240.0, 3360.0)	0.020
Margaric acid (17:0)	29.0 (24.0, 35.3)	29.0 (24.0, 35.1)	31.0 (25.4, 37.3)	0.003
Stearic acid (18:0)	644.0 (550.0, 760.0)	643.0 (549.0, 760.0)	653.0 (552.0, 769.0)	0.353
Arachidic acid (20:0)	23.0 (19.8, 26.6)	23.0 (19.9, 26.6)	22.0 (19.3, 26.6)	0.120
Docosanoic acid (22:0)	64.8 (55.0, 76.0)	65.0 (55.1, 76.0)	61.2 (49.3, 73.1)	< 0.001
Tricosanoic acid (23:0)	28.0 (23.3, 33.0)	28.0 (23.6, 33.0)	26.3 (21.0, 31.4)	< 0.001
Lignoceric acid (24:0)	55.0 (46.8, 65.0)	55.9 (47.0, 65.2)	51.0 (41.0, 62.5)	< 0.001

### Association between FFAs and all-cause mortality

In the USFA group, USFA positively associated with the all-cause mortality were myristoleic acid (14:1 n-5) (HR 1.02 [1.006–1.034]; *P* = 0.004), 16:1 n-7 (HR 1.001 [1.001–1.002]; *P* < 0.001), 18:1 n-7 (HR 1.006 [1.003–1.009]; *P* < 0.001), nervonic acid (24:1 n-9) (HR 1.007 [1.002–1.012]; *P* = 0.003), 20:3 n-9 (HR 1.027 [1.009–1.046]; *P* = 0.003), 22:4 n-6 (HR 1.024 [1.012–1.036]; *P* < 0.001), and 22:5 n-6 (HR 1.019 [1.006–1.032]; *P* = 0.005), while 22:6 n-3 had a statistically lower risk of all-cause mortality (HR 0.998 [0.996–0.999]; *P* = 0.007) (Model 4) (Table 3). Among the SFA group, 16:0 demonstrated a higher risk of all-cause mortality (HR 1.00 [1.00–1.00]; *P* = 0.022), while 23:0 (HR 0.975 [0.959–0.991]; *P* = 0.002) and 24:0 (HR 0.992 [0.984–0.999]; *P* = 0.036) were linked to a lower risk of all-cause mortality in the fully-adjusted model (Model 4) (Table 3). After adjusting for HOMA-IR, the findings did not change significantly (Supplementary Table S4).

**Table 3:** Univariate and multivariate Cox regression analysis of serum fatty acids associated with all-cause mortality

	Model 1		Model 2		Model 3		Model 4	
	HR (95%CI)	*P*	HR (95%CI)	*P*	HR (95%CI)	*P*	HR (95%CI)	*P*
Unsaturated fatty acids (*N* = 3719)								
Myristoleic acid (14:1 n-5)	1.006 (0.998–1.015)	0.134	1.013 (1.005–1.021)	0.002	1.006 (0.993–1.019)	0.373	1.020 (1.006–1.034)	0.004
Palmitoleic acid (16:1 n-7)	1.001 (1.000-1.001)	< 0.001	1.001 (1.000-1.001)	< 0.001	1.001 (1.000-1.001)	0.030	1.001 (1.001–1.002)	< 0.001
cis-Vaccenic acid (18:1 n-7)	1.002 (1.002–1.003)	0.002	1.003 (1.002–1.004)	< 0.001	1.003 (1.001–1.006)	0.013	1.006 (1.003–1.009)	< 0.001
Oleic acid (18:1 n-9)	1.000 (1.000–1.000)	< 0.001	1.000 (1.000–1.000)	0.005	1.000 (1.000–1.000)	0.908	1.000 (1.000–1.000)	0.153
Eicosenoic acid (20:1 n-9)	1.016 (1.005–1.027)	0.006	1.014 (1.001–1.028)	0.042	0.998 (0.973–1.024)	0.907	1.013 (0.987–1.039)	0.337
Nervonic acid (24:1 n-9)	1.002 (0.997–1.007)	0.388	1.002 (0.997–1.007)	0.368	1.002 (0.998–1.007)	0.294	1.007 (1.002–1.012)	0.003
Linoleic acid (18:2 n-6)	1.000 (1.000–1.000)	0.002	1.000 (1.000–1.000)	0.212	1.000 (1.000–1.000)	< 0.001	1.000 (1.000–1.000)	0.213
α-Linolenic acid (18:3 n-3)	1.000 (0.998–1.002)	0.791	1.000 (0.998–1.002)	0.776	0.996 (0.993-1.000)	0.027	0.998 (0.995–1.002)	0.294
γ-Linolenic acid (18:3 n-6)	1.002 (0.998–1.005)	0.334	1.002 (0.999–1.006)	0.211	0.999 (0.995–1.003)	0.749	0.999 (0.995–1.004)	0.800
Stearidonic acid (18:4 n-3)	1.015 (0.994–1.037)	0.165	1.013 (0.998–1.040)	0.308	0.990 (0.957–1.025)	0.577	0.998 (0.962–1.035)	0.912
Eicosadienoic acid (20:2 n-6)	1.007 (0.996–1.019)	0.185	1.009 (0.998–1.022)	0.181	0.995 (0.997–1.013)	0.578	1.013 (0.995–1.032)	0.166
Dihomo-γ-Linolenic acid (20:3 n-6)	0.999 (0.997–1.001)	0.174	1.000 (0.998–1.002)	0.982	0.998 (0.996–1.001)	0.148	1.000 (0.998–1.002)	0.947
Eicosatrienoic acid (20:3 n-9)	1.036 (1.024–1.048)	< 0.001	1.033 (1.018–1.049)	< 0.001	1.027 (1.010–1.045)	0.002	1.027 (1.009–1.046)	0.003
Arachidonic acid (20:4 n-6)	1.000 (1.000-1.001)	0.021	1.000 (1.000-1.001)	0.145	1.000 (1.000-1.001)	0.675	1.000 (1.000-1.001)	0.834
Eicosapentaenoic acid (20:5 n-3)	1.001 (0.999–1.002)	0.330	0.998 (0.997-1.000)	0.107	0.998 (0.996-1.000)	0.093	0.999 (0.997–1.001)	0.245
Docosatetraenoic acid (22:4 n-6)	1.013 (1.006–1.020)	< 0.001	1.020 (1.013–1.028)	< 0.001	1.021 (1.009–1.033)	< 0.001	1.024 (1.012–1.036)	< 0.001
Docosapentaenoic acid (22:5 n-3)	1.007 (1.003–1.012)	< 0.001	1.002 (0.997–1.007)	0.424	0.998 (0.992–1.004)	0.563	1.000 (0.995–1.006)	0.902
Docosapentaenoic acid (22:5 n-6)	1.009 (0.999–1.019)	0.089	1.019 (1.008–1.030)	< 0.001	1.013 (1.000-1.026)	0.045	1.019 (1.006–1.032)	0.005
Docosahexaenoic acid (22:6 n-3)	1.000 (0.999–1.001)	0.886	0.998 (0.996–0.999)	0.002	0.997 (0.996–0.999)	0.001	0.998 (0.996–0.999)	0.007
Saturated fatty acids (*N* = 3900)								
Capric acid (10:0)	1.012 (0.997–1.028)	0.120	1.027 (1.012–1.043)	< 0.001	1.017 (0.996–1.039)	0.114	1.020 (0.999–1.042)	0.056
Lauric acid (12:0)	1.000 (0.995–1.004)	0.913	1.002 (0.997–1.006)	0.448	0.998 (0.992–1.003)	0.435	1.000 (0.994–1.005)	0.956
Myristic acid (14:0)	1.000 (1.000-1.001)	0.321	1.001 (1.000-1.002)	0.034	1.000 (0.998–1.002)	0.910	1.002 (1.000-1.003)	0.111
Pentadecanoic acid (15:0)	1.004 (0.995–1.013)	0.354	1.005 (0.996–1.015)	0.290	0.996 (0.984–1.009)	0.589	1.008 (0.994–1.022)	0.251
Palmitic acid (16:0)	1.000 (1.000–1.000)	0.029	1.000 (1.000–1.000)	0.013	1.000 (1.000–1.000)	0.506	1.000 (1.000–1.000)	0.022
Margaric acid (17:0)	1.010 (1.001–1.019)	0.022	1.006 (0.996–1.016)	0.222	0.996 (0.982–1.011)	0.630	1.006 (0.991–1.022)	0.429
Stearic acid (18:0)	1.000 (1.000-1.001)	0.185	1.000 (1.000-1.001)	0.084	1.000 (0.999–1.001)	0.722	1.000 (1.000-1.001)	0.275
Arachidic acid (20:0)	0.987 (0.969–1.006)	0.176	0.993 (0.974–1.012)	0.475	0.978 (0.958–0.998)	0.033	0.992 (0.972–1.013)	0.468
Docosanoic acid (22:0)	0.983 (0.977–0.990)	< 0.001	0.990 (0.984–0.997)	0.005	0.988 (0.981–0.995)	< 0.001	0.994 (0.987-1.000)	0.060
Tricosanoic acid (23:0)	0.960 (0.945–0.974)	< 0.001	0.964 (0.949–0.980)	< 0.001	0.961 (0.945–0.977)	< 0.001	0.975 (0.959–0.991)	0.002
Lignoceric acid (24:0)	0.976 (0.968–0.984)	< 0.001	0.985 (0.977–0.993)	< 0.001	0.984 (0.976–0.992)	< 0.001	0.992 (0.984–0.999)	0.036

Unsaturated fatty acids: model 1 unadjusted; model 2 adjusted by age, gender, and education level; model 3 adjusted by model 2 and increasing exercise, reducing salt diet, protein, sugar, dietary fiber, and triglycerides; model 4 adjusted by model 3 and diabetes, hypertension, congestive heart failure, coronary heart disease, angina, heart attack, stroke, and cancer. Saturated fatty acids: model 1 unadjusted; model 2 adjusted by age, gender, and education level; model 3 adjusted by model 2 and increasing exercise, reducing salt diet, protein, carbohydrate, sugar, dietary fiber, total monounsaturated fatty acids, total polyunsaturated fatty acids, and triglycerides; model 4 adjusted by model 3 and diabetes, hypertension, congestive heart failure, coronary heart disease, angina, heart attack, stroke, and cancer. Abbreviations: HR, hazard ratio; CI, confidence interval

To explore the different risk of all-cause mortality in different FFAs concentration, we divided the FFAs into quartiles (Table 4). In USFA, comparing with Q1, 16:1 n-7, 18:1 n-7, 20:3 n-9, and 22:4 n-6 in Q3 and Q4 had significantly elevated risk of all-cause mortality (*P* < 0.05). In SFA, comparing with Q1, serum 23:0 and 24:0 in Q2, Q3 and Q4 had significantly reduced risk of all-cause mortality (*P* < 0.05).

**Table 4:** Association of quartile percentages of serum fatty acids with all-cause mortality in different gender population

All-cause mortality	All		Male		Female		*P* for interaction
	HR (95%CI)	*P*	HR (95%CI)	*P*	HR (95%CI)	*P*	
Unsaturated fatty acids (*N* = 3719)							
Myristoleic acid (14:1 n-5)							0.123
Q1 (< 3.34)	1 (ref)		1 (ref)		1 (ref)		
Q2 (3.34–5.99)	0.921 (0.674–1.260)	0.607	0.995 (0.682–1.451)	0.977	0.867 (0.495–1.518)	0.617	
Q3 (6.00-9.99)	1.101 (0.813–1.490)	0.534	0.973 (0.662–1.431)	0.889	1.451 (0.878–2.398)	0.146	
Q4 (≥ 10.00)	0.905 (0.662–1.236)	0.529	0.629 (0.414–0.956)	0.030	1.515 (0.921–2.491)	0.102	
Palmitoleic acid (16:1 n-7)							
Q1 (< 135)	1 (ref)		1 (ref)		1 (ref)		0.030
Q2 (135–198)	1.351 (0.964–1.895)	0.081	1.487 (1.008–2.195)	0.046	1.404 (0.707–2.787)	0.332	
Q3 (199–297)	1.507 (1.083–2.098)	0.015	1.213 (0.803–1.834)	0.359	2.707 (1.458–5.026)	0.002	
Q4 (≥ 298)	1.581 (1.140–2.193)	0.006	1.085 (0.716–1.645)	0.699	3.275 (1.779–6.030)	<0.001	
cis-Vaccenic acid (18:1 n-7)							
Q1 (< 115)	1 (ref)		1 (ref)		1 (ref)		<0.001
Q2 (115–140)	1.319 (0.926–1.878)	0.125	1.195 (0.787–1.814)	0.403	1.736 (0.884–3.410)	0.109	
Q3 (141–176)	1.488 (1.050–2.107)	0.025	1.240 (0.816–1.883)	0.313	2.273 (1.186–4.358)	0.013	
Q4 (≥ 177)	2.125 (1.555–2.978)	<0.001	1.216 (0.808–1.831)	0.348	5.113 (2.812–9.297)	<0.001	
Nervonic acid (24:1 n-9)							
Q1 (< 73.0)	1 (ref)		1 (ref)		1 (ref)		0.073
Q2 (73.0-86.2)	0.977 (0.712–1.340)	0.886	0.866 (0.596–1.259)	0.451	1.487 (0.801–2.759)	0.209	
Q3 (86.3-101.9)	0.997 (0.725–1.370)	0.985	0.986 (0.667–1.457)	0.943	1.471 (0.807–2.680)	0.208	
Q4 (≥ 102.0)	1.165 (0.861–1.577)	0.323	1.059 (0.706–1.588)	0.783	2.026 (1.159–3.539)	0.013	
Eicosatrienoic acid (20:3 n-9)							
Q1 (< 5.00)	1(ref)		1 (ref)		1 (ref)		0.318
Q2 (5.00-6.99)	1.207 (0.846–1.722)	0.300	1.332 (0.855–2.074)	0.205	0.937 (0.511–1.717)	0.833	
Q3 (7.00-9.44)	1.598 (1.140–2.240)	0.007	1.443 (0.931–2.236)	0.101	1.768 (1.040–3.006)	0.035	
Q4 (≥ 9.44)	2.044 (1.479–2.824)	<0.001	1.559 (1.020–2.385)	0.040	2.742 (1.663–4.521)	<0.001	
Docosatetraenoic acid (22:4 n-6)							
Q1 (< 20.0)	1 (ref)		1 (ref)		1 (ref)		0.004
Q2 (20.0-25.2)	1.180 (0.837–1.665)	0.344	1.117 (0.740–1.685)	0.598	1.280 (0.684–2.396)	0.440	
Q3 (25.3–32.4)	1.511 (1.086–2.102)	0.014	1.008 (0.664–1.530)	0.970	2.673 (1.522–4.696)	<0.001	
Q4 (≥ 32.5)	1.652 (1.195–2.285)	0.002	0.987 (0.656–1.487)	0.951	3.292 (1.891–5.734)	<0.001	
Docosapentaenoic acid (22:5 n-6)							
Q1 (< 15.0)	1 (ref)		1 (ref)		1 (ref)		0.427
Q2 (15.0-19.6)	0.863 (0.620–1.199)	0.379	0.945 (0.631–1.414)	0.783	0.794 (0.448–1.408)	0.430	
Q3 (19.7–25.8)	1.185 (0.873–1.608)	0.276	1.015 (0.688–1.496)	0.941	1.533 (0.928–2.534)	0.095	
Q4 (≥ 25.9)	1.190 (0.876–1.617)	0.265	1.068 (0.717–1.590)	0.748	1.519 (0.928–2.486)	0.097	
Docosahexaenoic acid (22:6 n-3)							
Q1 (< 109)	1 (ref)		1 (ref)		1 (ref)		0.233
Q2 (109–143)	0.820 (0.593–1.132)	0.227	0.750 (0.502–1.121)	0.161	1.121 (0.637–1.975)	0.692	
Q3 (144–198)	1.065 (0.790–1.437)	0.679	0.982 (0.677–1.425)	0.926	1.469 (0.864–2.498)	0.155	
Q4 (≥ 199)	0.970 (0.714–1.318)	0.846	0.936 (0.630–1.391)	0.745	1.354 (0.800-2.291)	0.259	
Saturated fatty acids (*N* = 3900)							
Palmitic acid (16:0)							
Q1 (< 2159)	1 (ref)		1 (ref)		1 (ref)		0.142
Q2 (2160–2629)	1.173 (0.856–1.607)	0.320	0.970 (0.667–1.410)	0.873	2.099 (1.123–3.921)	0.020	
Q3 (2630–3229)	1.184 (0.865–1.621)	0.291	0.785 (0.530–1.162)	0.227	2.801 (1.533–5.121)	<0.001	
Q4 (≥ 3230)	1.331 (0.979–1.808)	0.068	0.764 (0.518–1.126)	0.174	3.609 (1.998–6.520)	<0.001	
Tricosanoic acid (23:0)							
Q1 (< 23.3)	1(ref)		1(ref)		1 (ref)		0.620
Q2 (23.3–27.9)	0.691 (0.513–0.930)	0.015	0.461 (0.310–0.684)	<0.001	0.807 (0.474–1.376)	0.432	
Q3 (28-32.9)	0.730 (0.539–0.989)	0.042	0.542 (0.354–0.830)	0.005	0.947 (0.581–1.543)	0.826	
Q4 (≥ 33.0)	0.724 (0.528–0.992)	0.045	0.572 (0.402–0.815)	0.002	0.852 (0.531–1.368)	0.507	
Lignoceric acid (24:0)							
Q1 (< 46.8)	1 (ref)		1 (ref)		1 (ref)		0.170
Q2 (46.8–54.9)	0.705 (0.534–0.931)	0.014	0.500 (0.348–0.718)	<0.001	1.295 (0.814–2.062)	0.275	
Q3 (55.0-64.9)	0.451 (0.332–0.613)	<0.001	0.333 (0.224–0.496)	<0.001	0.792 (0.477–1.316)	0.368	
Q4 (≥ 65.0)	0.569 (0.427–0.758)	<0.001	0.365 (0.246–0.541)	<0.001	1.153 (0.726–1.831)	0.546	

Abbreviations Q1, below 25th percentile; Q2, between 25th percentile and 50th percentile; Q3, between 50th percentile and 75th percentile; Q4, above 75th percentile; HR, hazard ratio; CI, confidence interval; ref, reference

**Fig. 2:**
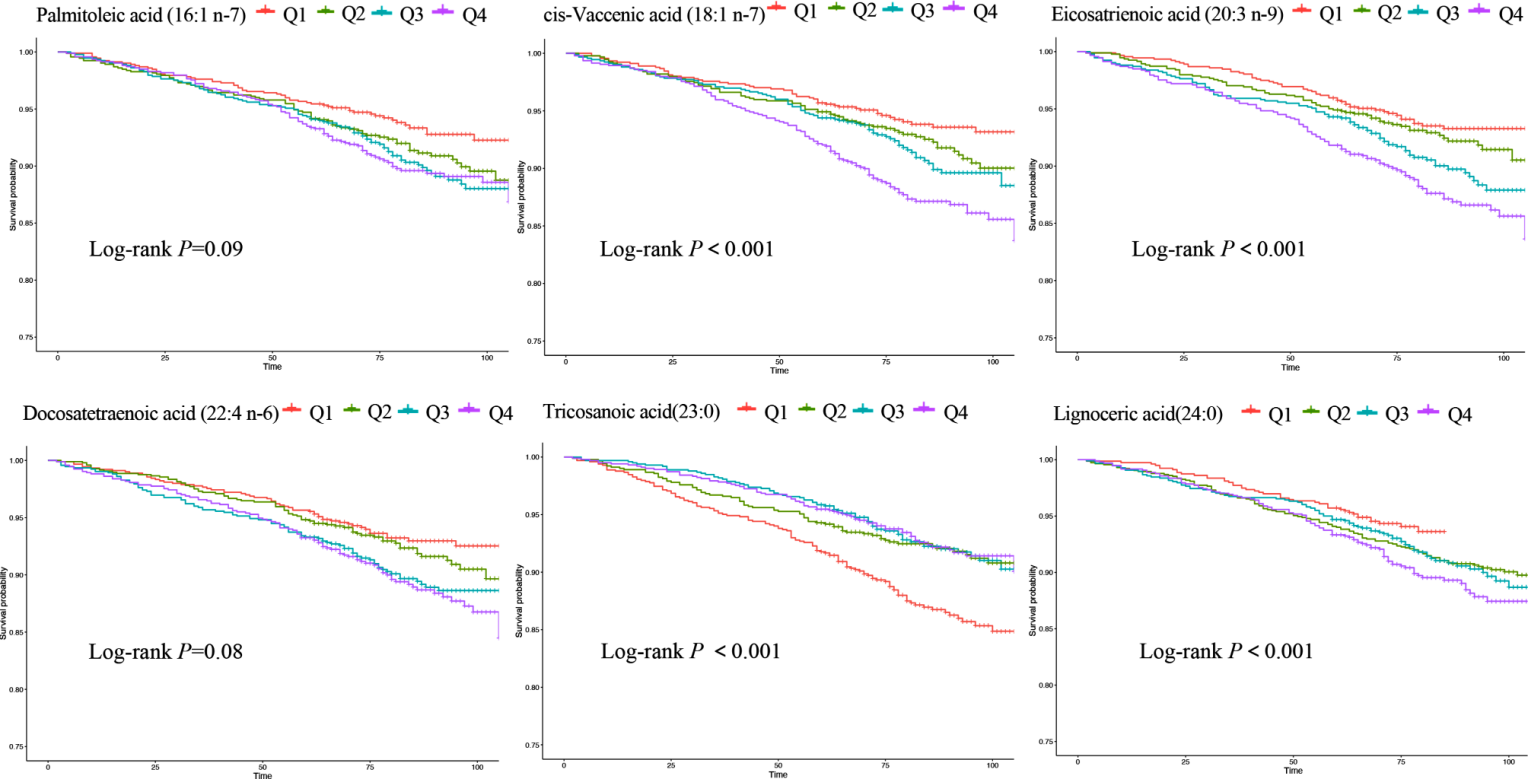
Kaplan–Meier survival curves for all-cause mortality according to serum fatty acids quartiles. Q1, below 25th percentile; Q2, between 25th percentile and 50th percentile; Q3, between 50th percentile and 75th percentile; Q4, above 75th percentile

Kaplan–Meier survival curves for all-cause mortality were shown in Fig. 2, which displayed similar trends as in Cox regression. However, subgroup analysis in USFA, indicated only 16:1 n-7, 18:1 n-7 and 22:4 n-6, had interactions with gender. Meanwhile, in the SFA group, no interaction between FFA and gender was found. (Table 4).

**Fig. 3:**
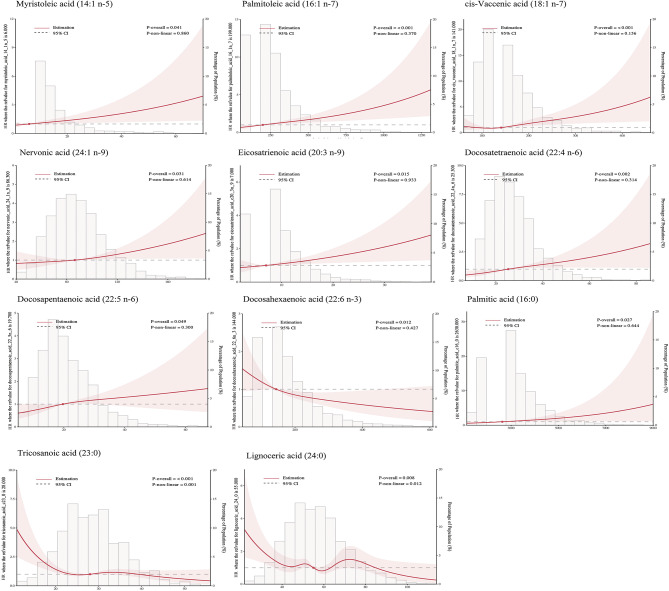
Restricted Cubic Spline analysis for association of serum fatty acids with all-cause mortality

To evaluate the non-linear relationship between FFAs and all-cause mortality, we used RCS analysis. In the USFA group, serum 14:1 n-5 (*P*-linear = 0.041), 16:1 n-7 (*P*-linear < 0.001), 18:1 n-7 (*P*-linear < 0.001), 24:1 n-9 (*P*-linear = 0.031), 20:3 n-9 (*P*-linear = 0.015), 22:4 n-6 (*P*-linear = 0.002), and 22:5 n-6 (*P*-linear = 0.049) were linearly associated with higher risks of all-cause mortality, while, 22:6 n-3 were linearly associated with negative risk of all-cause mortality (*P*-linear = 0.012) (Fig. 3). In the SFA group, 16:0 (*P*-linear = 0.027) was linearly associated with higher risk of all-cause mortality, and the relationship between serum 23:0, 24:0 and risk of all-cause mortality showed L-shaped correlation curve which indicated that the lower concentration association with a higher all-cause mortality risk (Fig. 3).

### FFAs and cardiovascular mortality

Unlike the results between FFAs and all-cause mortality, in the USFA group, only 22:6 n-3 (HR 0.997 [0.994-1.000]; *P* = 0.035) had significant negative cardiovascular mortality risk while none of the SFA was found to be associated with cardiovascular mortality (Table 5). After adjusting for HOMA-IR, the findings did not change significantly (Supplementary Table S4). While, comparing with Q1, the level of 22:6 n-3 in Q2, Q3 and Q4 had no significantly associated with risk of cardiovascular mortality (Supplementary Table S5). The relationship between USFA, 22:6 n-3, and cardiovascular mortality was characterized by neither a linear nor nonlinear association (*P*-linear = 0.215; *P*-nonlinear = 0.260) (Supplementary Figure S1).

**Table 5:** Univariate and multivariate Cox regression analysis of serum fatty acids and associated with cardiovascular mortality

	Model 1		Model 2		Model 3		Model 4	
	HR (95%CI)	*P*	HR (95%CI)	*P*	HR (95%CI)	*P*	HR (95%CI)	*P*
Unsaturated fatty acids (*N* = 3719)								
Myristoleic acid (14:1 n-5)	0.999 (0.977–1.022)	0.936	1.007 (0.986–1.029)	0.516	1.007 (0.985–1.030)	0.534	1.006 (0.984–1.029)	0.581
Palmitoleic acid (16:1 n-7)	1.000 (0.999–1.001)	0.457	1.000 (0.999–1.001)	0.541	1.000 (0.999–1.002)	0.528	1.000 (0.999–1.001)	0.603
cis-Vaccenic acid (18:1 n-7)	1.002 (1.000-1.004)	0.043	1.002 (0.999–1.005)	0.225	1.002 (0.999–1.005)	0.196	1.001 (0.998–1.005)	0.484
Oleic acid (18:1 n-9)	1.000 (1.000–1.000)	0.143	1.000 (1.000–1.000)	0.271	1.000 (1.000–1.000)	0.263	1.000 (1.000–1.000)	0.812
Eicosenoic acid (20:1 n-9)	1.011 (0.986–1.035)	0.394	1.009 (0.979–1.041)	0.555	1.010 (0.979–1.042)	0.535	0.993 (0.959–1.027)	0.668
Nervonic acid (24:1 n-9)	1.000 (0.990–1.009)	0.921	0.997 (0.998–1.006)	0.457	0.997 (0.988–1.006)	0.453	1.004 (0.996–1.013)	0.336
Linoleic acid (18:2 n-6)	1.000 (1.000–1.000)	0.215	1.000 (1.000–1.000)	0.773	1.000 (1.000–1.000)	0.817	1.000 (1.000–1.000)	0.863
α-Linolenic acid (18:3 n-3)	1.000 (0.996–1.004)	0.977	1.000 (0.996–1.004)	0.990	1.000 (0.996–1.004)	0.989	0.999 (0.995–1.003)	0.528
γ-Linolenic acid (18:3 n-6)	1.001 (0.995–1.008)	0.661	1.001 (0.994–1.008)	0.833	1.000 (0.993–1.007)	0.947	0.997 (0.990–1.004)	0.407
Stearidonic acid (18:4 n-3)	1.015 (0.972–1.059)	0.502	1.008 (0.952–1.067)	0.783	1.006 (0.948–1.068)	0.832	0.975 (0.909–1.047)	0.493
Eicosadienoic acid (20:2 n-6)	1.009 (0.988–1.031)	0.412	1.008 (0.982–1.035)	0.554	1.007 (0.981–1.034)	0.604	1.006 (0.980–1.032)	0.674
Dihomo-γ-Linolenic acid (20:3 n-6)	0.997 (0.993–1.001)	0.157	0.998 (0.994–1.002)	0.246	0.997 (0.993–1.001)	0.193	0.998 (0.993–1.002)	0.296
Eicosatrienoic_acid (20:3 n-9)	1.031 (1.005–1.059)	0.021	1.021 (0.985–1.057)	0.252	1.020 (0.984–1.056)	0.281	1.003 (0.965–1.043)	0.866
Arachidonic_acid (20:4 n-6)	1.001 (1.000-1.002)	0.034	1.000 (1.000-1.001)	0.260	1.000 (1.000-1.001)	0.330	1.000 (0.999–1.001)	0.605
Eicosapentaenoic acid (20:5 n-3)	1.001 (0.998–1.004)	0.613	0.998 (0.994–1.002)	0.300	0.998 (0.994–1.002)	0.257	0.998 (0.994–1.002)	0.261
Docosatetraenoic acid (22:4 n-6)	1.014 (1.002–1.028)	0.027	1.022 (1.008–1.037)	0.003	1.023 (1.007–1.038)	0.004	1.015 (0.998–1.031)	0.079
Docosapentaenoic acid (22:5 n-3)	1.009 (1.000-1.017)	0.042	1.002 (0.993–1.011)	0.666	1.002 (0.992–1.011)	0.712	0.999 (0.991–1.008)	0.909
Docosapentaenoic acid (22:5 n-6)	1.009 (0.988–1.029)	0.414	1.013 (0.991–1.035)	0.253	1.012 (0.989–1.035)	0.306	1.007 (0.985–1.031)	0.528
Docosahexaenoic acid (22:6 n-3)	1.000 (0.997–1.002)	0.752	0.997 (0.994-1.000)	0.026	0.996 (0.994–0.999)	0.017	0.997 (0.994–0.999)	0.035
Saturated fatty acids (*N* = 3900)								
Capric acid (10:0)	1.009 (0.976–1.043)	0.594	1.030 (0.997–1.064)	0.075	1.029 (0.996–1.064)	0.087	1.025 (0.992–1.059)	0.134
Lauric acid (12:0)	1.000 (0.992–1.008)	0.952	1.002 (0.994–1.011)	0.592	1.002 (0.994–1.010)	0.635	1.003 (0.994–1.011)	0.547
Myristic acid (14:0)	1.001 (0.999–1.002)	0.522	1.001 (0.999–1.003)	0.148	1.001 (0.999–1.003)	0.194	1.001 (0.999–1.003)	0.419
Pentadecanoic acid (15:0)	1.004 (0.987–1.021)	0.649	1.006 (0.988–1.025)	0.521	1.005 (0.987–1.024)	0.593	1.005 (0.988–1.022)	0.573
Palmitic acid (16:0)	1.000 (1.000–1.000)	0.213	1.000 (1.000–1.000)	0.148	1.000 (1.000–1.000)	0.173	1.000 (1.000–1.000)	0.504
Margaric acid (17:0)	1.016 (1.001–1.031)	0.041	1.013 (0.995–1.030)	0.158	1.012 (0.994–1.029)	0.194	1.007 (0.991–1.024)	0.390
Stearic acid (18:0)	1.000 (0.999–1.001)	0.745	1.000 (0.999–1.001)	0.610	1.000 (0.999–1.001)	0.665	1.000 (0.999–1.001)	0.812
Arachidic acid (20:0)	0.968 (0.931–1.006)	0.094	0.977 (0.939–1.017)	0.251	0.976 (0.938–1.016)	0.234	0.992 (0.957–1.028)	0.650
Docosanoic acid (22:0)	0.975 (0.962–0.989)	<0.001	0.985 (0.972–0.999)	0.036	0.985 (0.972–0.999)	0.035	0.994 (0.981–1.007)	0.350
Tricosanoic acid (23:0)	0.961 (0.933–0.990)	0.009	0.970 (0.940–1.001)	0.057	0.970 (0.940–1.001)	0.056	0.988 (0.958–1.019)	0.445
Lignoceric acid (24:0)	0.965 (0.949–0.981)	<0.001	0.978 (0.963–0.994)	0.006	0.979 (0.963–0.994)	0.008	0.989 (0.974–1.005)	0.175

Unsaturated fatty acids: model 1 unadjusted; model 2 adjusted by age and education level; model 3 adjusted by model 2 and abdominal obesity, and reducing salt diet; model 4 adjusted by model 3 and diabetes, hypertension, congestive heart failure, coronary heart disease, angina, heart attack, stroke, and cancer. Saturated fatty acids: model 1 unadjusted; model 2 adjusted by age, gender, and education level; model 3 adjusted by model 2 and abdominal obesity, reducing salt diet, and triglycerides; model 4 adjusted by model 3 and diabetes, hypertension, congestive heart failure, coronary heart disease, angina, heart attack, stroke, and cancer. Abbreviations: HR, hazard ratio; CI, confidence interval

## Discussion

In this prospective cohort study utilizing data from NHANES, our results highlight the influence of various circulating FFAs on all-cause and cardiovascular mortality. Additionally, the risk associated with all-cause and cardiovascular mortality exhibited significant variations across different FFAs. Specifically, USFA, serum 22:6 n-3 demonstrated a reduced risk in terms of both all-cause and cardiovascular mortality. Among SFA, lower concentrations of circulating 23:0 and 24:0 exhibited higher risk of all-cause mortality.

It is widely accepted that USFA are associated with protective effects on human health, in contrast to the recognized adverse impact of SFA on overall well-being and longevity. Dietary recommendations have emphasized on the reduction of SFA [33], yet the evidence remains inconclusive. Meta-analyses have yielded conflicting findings, with some indicating no association between SFA intake and risk of CAD [34]. Research has shown that diverse types of SFA, distinguished by variations in carbon bond length and parity, demonstrate different effects on cardiovascular risk factors [35]. The observed heterogeneity of effects across the circulating composition of specific SFA, may be attributed to the interplay of dietary intake and endogenous metabolism and synthesis [36]. This is indirectly supported by the positive yet nonsignificant associations observed for the circulating concentration of 16:0 and 18:0 which are synthesized in the body and only weakly with CAD [34]. Moreover, 16:0 may raise cholesterol levels to greater extends than 18:0 [4]; substituting 16:0 with 18:0 could lower LDL concentrations [37]. Remarkably, a recent study emphasized that, across the entire population, individuals exhibiting higher serum levels of very long-chain SFAs (specifically 22:0 and 24:0) as a proportion of total serum FFAs, experienced a reduction in risks associated with all-cause mortality, CHD, and CVD mortality [38]. In our study, we observed a similar association between 24:0 and all-cause mortality, but not 22:0. Notably, we identified another very long-chain SFA, 23:0, which was linked to reduced all-cause mortality risk. Given that the highest total very long-chain SFA are primarily derived from the intake of peanuts and macadamia nuts [39], we found a significant negative association with all-cause mortality, with a HR of 0.78 (95% CI 0.76–0.81) [40]. The EPIC-InterAct case-cohort study revealed an inverse association between incident type 2 diabetes and plasma levels of long-chain SFAs (23:0 and 24:0); a finding that remained robust across various sensitivity analyses [41]. In a previous cohort study, replacing SFA, particularly 16:0 and 18:0, with plant proteins led to decreased risk of myocardial infarction [42]. However, no risk reduction was observed when SFA were substituted with MUFA or PUFA [42].

A meta-analysis revealed that the consumption of omega-3 fatty acids, encompassing 20:5 n-3 (EPA), DHA, and plant-derived 18:3 n-3 (ALA), is linked to a decreased risk of all-cause mortality, cardiovascular mortality, and CVD events among individuals with atherosclerotic cardiovascular disease [43]. In contrast, no significant association was observed between the omega-6 intake or total PUFA and these events [43]. Though guidelines recommend the augmentation of omega-3 fatty acids, recent trials have not confirmed this [44]; increase in omega-3 intake demonstrates minimal impact on both all-cause mortality and cardiovascular mortality [44]. Additionally, omega-3 fatty acids supplementation may also not demonstrate significantly beneficial influence on cancer incidence, non-vascular mortality, or all-cause mortality [45].

In our study, only serum DHA (22:6 n-3) was associated with a lower risk of all-cause mortality, and cardiovascular mortality. Conversely, 18:2 n-6 (LA), ALA, DHA (22:4 n-6), DHA (22:5 n-6), and EPA were associated with an increased risk of all-cause mortality. These diverse outcomes could be attributed, in part, to variations in participant characteristics and the detection methods for circulating fatty acids, influencing the associations between self-reported intake and actual circulating levels of EPA and DHA [46]. Another source of variability among different studies could be attributable to the normalization process. Indeed, circulating fatty acids are often expressed as a percentage of total measured fatty acids [47–49]. As an example, Johnson and colleagues reported both absolute and percentage normalization for free fatty acids showing how different normalization approaches led to different results [50]. Factors such as BMI, alcohol intake, and the method of expressing circulating fatty acids may play an important role in shaping the associations between dietary and circulating FFAs [46]. Furthermore, long-term dietary habits influenced the fatty acid composition of adipose tissue, which is largely reflected in circulating FFAs [51]. Specific dietary factors (e.g. low intake of whole grains; high intake of refined carbohydrates or trans fat) significantly reduce metabolic stress, and lowering risk of cardiovascular events [52]. It is noteworthy that dietary intake was commonly assessed using food frequency questionnaires in epidemiological studies, a method that may generate approximately 50% inaccurate data [53]. Epidemiological investigations examining the association between blood levels of EPA and DHA with clinical events could reveal more robust correlations compared to studies assessing solely dietary intake [54, 55]. Nevertheless, as showed in our study, there are significant interactions between USFA (16:1 n-7, 18:1 n-7 and 22:4 n-6) and gender in terms of all-cause mortality. Gender influences circulating FFA levels due to differences in hormone levels, body fat percentage, and body fat distribution between males and females, which may further mediate the relationship between FFA and health outcomes [56, 57]. Therefore, future studies might consider sex-specific factors when assessing the mortality risk using FFA biomarkers.

### Strengths and limitations

To our knowledge, this is the first study to examine how different FFAs associate with all-cause mortality and cardiovascular mortality in a large, diverse, nationally representative sample of adults.

Meanwhile, this study has many limitations. The disparities in the origins of individual FFAs, whether from dietary intake or endogenous metabolism, is unknown, which may have an impact on FFAs levels in blood serum, so the relationship between levels of FFAs and all-cause and cardiovascular mortality needs to be further verified. Then, we could not determine the exact impact of various FFAs and concentrations on overall mortality. Thirdly, only one baseline value was considered, and the individual FFAs levels and distribution may change over years. And the level of circulating FFAs is mainly affected by various factors, such as the lipolytic activity of the adipose tissue stores, obesity, visceral fat, insulin resistance etc., even we have adjustment for BMI, waist circumference, diabetes, HOMA-IR, the relationship between levels of FFAs and CVD end-points need more evidence.

## Conclusions

In this nationally representative cohort of US adults, the different FFAs exhibited significant associations with risk of all-cause mortality. Achieving optimal concentrations of specific FFAs effectively lowered this risk of all-cause mortality, but this benefit was not observed in regards to cardiovascular mortality.

## Electronic supplementary material

Below is the link to the electronic supplementary material.

Supplementary Material 1

## Data Availability

The data included in this study are publicly and freely available without restriction at:https://wwwn.cdc.gov/nchs/nhanes/continuousnhanes.

## Abbreviations

SFA: Saturated fatty acids
USFA: Unsaturated fatty acids
MUSFA: Monounsaturated fatty acids
PUSFA: Polyunsaturated fatty acids
CVD: Cardiovascular disease
FFAs: Free fatty acids
CAD: Coronary artery disease
CHD: Coronary heart disease
NHANES: National Health and Nutrition Examination Survey
NCHS: National Center for Health Statistics
CDC: Centers for Disease Control and Prevention
FIP: Family income to poverty ratio
BMI: Body mass index
HR: Hazard ratio
CI: Confidence interval
RCS: Restricted cubic spline
HOMA-IR: Homeostatic model assessment for insulin resistance
CHF: Chronic heart failure
