# Supplementary material for: Free fatty acids and mortality among adults in the United States: a report from US National Health and Nutrition Examination Survey (NHANES)
Source: Nutr Metab (Lond). 2024 Sep 10;21:72. doi: 10.1186/s12986-024-00844-6 (PMC11389384; doi:10.1186/s12986-024-00844-6)
Supplement: Supplementary file 1 — Supplementary Material 1 [file 12986_2024_844_MOESM1_ESM.docx]

Supplementary Table S1. Univariate logistic regression for all-cause mortality.

| Variables | Unsaturated fatty acids (N=3719) | | Saturated fatty acid (N=3900) | |
| --- | --- | --- | --- | --- |
|  | HR (95% CI) | *P* | HR (95% CI) | *P* |
| Age | 9.78 (7.71-12.4) | <0.001 | 9.76 (7.74-12.3) | <0.001 |
| Gender | 0.62 (0.50-0.77) | <0.001 | 0.64 (0.52-0.80) | <0.001 |
| Race | 0.94 (0.87-1.01) | 0.092 | 0.96 (0.89-1.03) | 0.281 |
| Education level | 0.78 (0.69-0.89) | <0.001 | 0.75 (0.66-0.85) | <0.001 |
| FIP | 0.94 (0.84-1.04) | 0.230 | 0.92 (0.82-1.02) | 0.099 |
| Alcohol drinking | 0.93 (0.73-1.19) | 0.571 | 0.95 (0.75-1.20) | 0.656 |
| BMI | 0.90 (0.78-1.02) | 0.106 | 0.91 (0.80-1.04) | 0.164 |
| Abdominal obesity | 1.20 (0.96-1.50) | 0.108 | 1.14 (0.92-1.41) | 0.235 |
| Losing weight | 0.88 (0.71-1.10) | 0.262 | 0.86 (0.70-1.07) | 0.178 |
| Increasing exercise | 0.67 (0.54-0.83) | <0.001 | 0.62 (0.50-0.77) | <0.001 |
| Reducing salt diet | 1.44 (1.15-1.80) | 0.001 | 1.45 (1.17-1.80) | <0.001 |
| Reducing fat diet | 0.92 (0.74-1.15) | 0.466 | 0.95 (0.77-1.17) | 0.633 |
| Energy | 1.00 (1.00-1.00) | <0.001 | 1.00 (1.00-1.00) | <0.001 |
| Protein | 0.99 (0.99-1.00) | <0.001 | 0.99 (0.99-1.00) | <0.001 |
| Carbohydrate | 1.00 (1.00-1.00) | <0.001 | 1.00 (1.00-1.00) | <0.001 |
| Sugar | 1.00 (1.00-1.00) | 0.026 | 1.00 (1.00-1.00) | 0.029 |
| Dietary fiber | 0.99 (0.97-1.00) | 0.015 | 0.98 (0.97-1.00) | 0.004 |
| Total fat | 1.00 (1.00-1.00) | 0.056 | 1.00 (1.00-1.00) | 0.031 |
| Total SFA | 1.00 (0.99-1.00) | 0.172 | 1.00 (0.99-1.00) | 0.144 |
| Total MUFA | 0.99 (0.99-1.00) | 0.061 | 0.99 (0.99-1.00) | 0.033 |
| Total PUFA | 0.99 (0.98-1.00) | 0.030 | 0.99 (0.98-1.00) | 0.026 |
| Total cholesterol | 0.99 (0.99-1.00) | <0.001 | 1.00 (0.99-1.00) | 0.009 |
| HDL-C | 1.00 (0.99-1.00) | 0.452 | 1.00 (1.00-1.01) | 0.596 |
| LDL-C | 0.99 (0.99-1.00) | <0.001 | 0.99 (0.99-1.00) | <0.001 |
| Triglycerides | 1.00 (1.00-1.00) | 0.003 | 1.00 (1.00-1.00) | 0.005 |
| Diabetes | 3.63 (2.86-4.60) | <0.001 | 3.51 (2.79-4.42) | <0.001 |
| Hypertension | 4.56 (3.58-5.80) | <0.001 | 4.81 (3.81-6.09) | <0.001 |
| CHF | 5.83 (4.33-7.85) | <0.001 | 5.82 (4.35-7.79) | <0.001 |
| CHD | 6.56 (4.93-8.74) | <0.001 | 6.55 (4.96-8.63) | <0.001 |
| Angina | 4.06 (2.77-5.94) | <0.001 | 4.18 (2.91-6.02) | <0.001 |
| Heart attack | 5.14 (3.78-6.98) | <0.001 | 4.91 (3.65-6.61) | <0.001 |
| Stroke | 4.86 (3.57-6.60) | <0.001 | 5.00 (3.71-6.73) | <0.001 |
| Cancer | 3.80 (2.96-4.88) | <0.001 | 3.80 (2.99-4.85) | <0.001 |

Abbreviations: FIP, family income to poverty ratio; BMI, body mass index; SFA, saturated fatty acids; MUFA, monounsaturated fatty acids; PUFA, polyunsaturated fatty acids; HDL-C, high-density lipoprotein cholesterol; LDL-C, low-density lipoprotein cholesterol; CHF, congestive Heart Failure; CHD, coronary heart disease; HR, hazard ratio; CI, confidence interval.

Supplementary Table S2. Univariate logistic regression for cardiovascular mortality.

| Variables | Unsaturated fatty acids (N=3719) | | Saturated fatty acid (N=3900) | |
| --- | --- | --- | --- | --- |
|  | HR (95% CI) | *P* | HR (95% CI) | *P* |
| Age | 16.34 (9.59-27.86) | <0.001 | 15.59 (9.40-25.86) | 0.001 |
| Gender | 0.71 (0.46-1.09) | 0.116 | 0.63 (0.42-0.96) | 0.031 |
| Race | 0.95 (0.82-1.10) | 0.480 | 0.95 (0.83-1.10) | 0.515 |
| Education level | 0.62 (0.48-0.79) | <0.001 | 0.55 (0.43-0.70) | <0.001 |
| FIP | 0.84 (0.69-1.03) | 0.102 | 0.84 (0.69-1.02) | 0.081 |
| Alcohol drinking | 0.67 (0.43-1.04) | 0.075 | 0.72 (0.47-1.11) | 0.134 |
| BMI | 1.00 (0.77-1.30) | 0.997 | 1.09 (0.85-1.40) | 0.512 |
| Abdominal obesity | 1.60 (1.02-2.52) | 0.040 | 1.56 (1.02-2.40) | 0.041 |
| Losing weight | 1.16 (0.74-1.81) | 0.526 | 1.07 (0.70-1.64) | 0.749 |
| Increasing exercise | 0.88 (0.57-1.35) | 0.549 | 0.75 (0.50-1.13) | 0.171 |
| Reducing salt diet | 2.49 (1.54-4.02) | <0.001 | 2.48 (1.56-3.92) | <0.001 |
| Reducing fat diet | 1.24 (0.80-1.92) | 0.339 | 1.33 (0.87-2.03) | 0.186 |
| Energy | 1.00 (1.00-1.00) | 0.128 | 1.00 (1.00-1.00) | 0.242 |
| Protein | 1.00 (0.99-1.00) | 0.198 | 1.00 (0.99-1.00) | 0.372 |
| Carbohydrate | 1.00 (1.00-1.00) | 0.025 | 1.00 (1.00-1.00) | 0.014 |
| Sugar | 1.00 (1.00-1.00) | 0.199 | 1.00 (0.99-1.00) | 0.160 |
| Dietary fiber | 0.99 (0.96-1.01) | 0.190 | 0.99 (0.97-1.01) | 0.225 |
| Total fat | 1.00 (1.00-1.01) | 0.970 | 1.00 (1.00-1.01) | 0.385 |
| Total SFA | 1.00 (0.99-1.02) | 0.718 | 1.01 (0.99-1.02) | 0.368 |
| Total MUFA | 1.00 (0.99-1.01) | 0.970 | 1.01 (0.99-1.02) | 0.400 |
| Total PUFA | 1.00 (0.98-1.01) | 0.650 | 1.01 (0.99-1.02) | 0.472 |
| Total cholesterol | 1.00 (0.99-1.00) | 0.144 | 1.00 (0.99-1.00) | 0.068 |
| HDL-C | 0.99 (0.98-1.01) | 0.325 | 0.99 (0.97-1.00) | 0.093 |
| LDL-C | 0.99 (0.99-1.00) | 0.038 | 0.99 (0.99-1.00) | 0.012 |
| Triglycerides | 1.00 (1.00-1.00) | 0.141 | 1.00 (1.00-1.00) | 0.021 |
| Diabetes | 5.93 (3.84-9.18) | <0.001 | 6.34 (4.19-9.59) | <0.001 |
| Hypertension | 5.22 (3.21-8.50) | <0.001 | 4.71 (3.01-7.47) | <0.001 |
| CHF | 11.01 (6.75-17.94) | <0.001 | 11.23 (7.04-17.92) | <0.001 |
| CHD | 8.95 (5.36-14.95) | <0.001 | 7.41 (4.42-12.42) | <0.001 |
| Angina | 3.19 (1.39-7.33) | 0.006 | 2.88 (1.26-6.58) | 0.012 |
| Heart attack | 9.83 (5.99-16.13) | <0.001 | 7.38 (4.45-12.24) | <0.001 |
| Stroke | 5.60 (3.15-9.96) | <0.001 | 4.33 (2.36-7.95) | <0.001 |
| Cancer | 2.23 (1.25-3.96) | 0.006 | 2.16 (1.24-3.77) | 0.006 |

Abbreviations: FIP, family income to poverty ratio; BMI, body mass index; SFA, saturated fatty acids; MUFA, monounsaturated fatty acids; PUFA, polyunsaturated fatty acids; HDL- C, high-density lipoprotein cholesterol; LDL-C, low-density lipoprotein cholesterol; CHF, congestive Heart Failure; CHD, coronary heart disease; HR, hazard ratio; CI, confidence interval.

Supplementary Table S3. Variance inflation coefficient of serum fatty acids for all-cause and cardiovascular mortality.

| Unsaturated fatty acids (N=3719) | | | | Saturated fatty acid (N=3900) | | | |
| --- | --- | --- | --- | --- | --- | --- | --- |
| All-cause mortality | | Cardiovascular mortality | | All-cause mortality | | Cardiovascular mortality | |
| Variables | VIF | Variables | VIF | Variables | VIF | Variables | VIF |
| Age | 1.432 | Age | 1.340 | Age | 1.411 | Age | 1.362 |
| Gender | 1.496 | Education level | 1.269 | Gender | 1.531 | Gender | 1.527 |
| Education level | 1.300 | abdominal obesity | 2.564 | Education level | 1.286 | Education level | 1.278 |
| Increasing exercise | 1.441 | Reducing salt diet | 1.760 | Increasing exercise | 1.460 | Abdominal obesity | 2.603 |
| Reducing salt diet | 1.760 | Carbohydrate | 19.626 | Reducing salt diet | 1.809 | Reducing salt diet | 1.627 |
| Energy | 25.560 | LDL-C | 36.829 | Energy | 19.514 | Carbohydrate | 18.926 |
| Protein | 3.541 | Diabetes | 1.177 | Protein | 3.128 | LDL-C | 39.556 |
| Carbohydrate | 19.808 | Hypertension | 1.314 | Carbohydrate | 6.331 | Triglycerides | 5.486 |
| Sugar | 6.418 | CHF | 1.354 | Sugar | 4.675 | Diabetes | 1.173 |
| Dietary fiber | 2.286 | CHD | 1.000 | Dietary fiber | 2.177 | Hypertension | 1.321 |
| Total fat | 368.125 | Angina | 1.285 | Total fat | 424.090 | CHF | 1.329 |
| Total MUFA | 61.649 | Heart attack | 1.342 | Total MUFA | 7.151 | CHD | 1.557 |
| Total PUFA | 30.152 | Stroke | 1.161 | Total PUFA | 3.586 | Angina | 1.376 |
| Total cholesterol | 23.249 | Cancer | 1.107 | Total cholesterol | 36.749 | Heart attack | 1.437 |
| LDL-C | 18.274 |  |  | LDL-C | 27.470 | Stroke | 1.132 |
| Triglycerides | 2.873 |  |  | Triglycerides | 4.298 | Cancer | 1.108 |
| Diabetes | 1.319 |  |  | Diabetes | 1.312 |  |  |
| Hypertension | 1.284 |  |  | Hypertension | 1.327 |  |  |
| CHF | 1.387 |  |  | CHF | 1.350 |  |  |
| CHD | 1.737 |  |  | CHD | 1.669 |  |  |
| Angina | 1.506 |  |  | Angina | 1.455 |  |  |
| Heart attack | 1.745 |  |  | Heart attack | 1.522 |  |  |
| Stroke | 1.194 |  |  | Stroke | 1.177 |  |  |
| Cancer | 1.117 |  |  | Cancer | 1.125 |  |  |

Abbreviations: VIF, variance inflation coefficient; LDL-C, low-density lipoprotein cholesterol; CHF, congestive Heart Failure; CHD, coronary heart disease; MUFA, monounsaturated fatty acids; PUFA, polyunsaturated fatty acids.

Supplementary Table S4. Sensitivity analysis adjusting for whole body insulin resistance

|  | All-cause mortality | | Cardiovascular mortality | |
| --- | --- | --- | --- | --- |
|  | HR (95%CI) | *P* | HR (95%CI) | *P* |
| Unsaturated fatty acids (N=3719) | | | | |
| Myristoleic acid (14:1 n-5) | 1.021 (1.007-1.035) | 0.003 | 1.005 (0.982-1.029) | 0.660 |
| Palmitoleic acid (16:1 n-7) | 1.001 (1.001-1.002) | <0.001 | 1.000 (0.999-1.001) | 0.739 |
| cis-Vaccenic acid (18:1 n-7) | 1.006 (1.003-1.009) | <0.001 | 1.001 (0.997-1.004) | 0.594 |
| Oleic acid (18:1 n-9) | 1.000 (1.000-1.000) | 0.145 | 1.000 (1.000-1.000) | 0.664 |
| Eicosenoic acid (20:1 n-9) | 1.014 (0.988-1.041) | 0.297 | 0.990 (0.956-1.025) | 0.570 |
| Nervonic acid (24:1 n-9) | 1.007 (1.002-1.012) | 0.003 | 1.004 (0.995-1.013) | 0.340 |
| Linoleic acid (18:2 n-6) | 1.000 (1.000-1.000) | 0.254 | 1.000 (1.000-1.000) | 0.954 |
| α-Linolenic acid (18:3 n-3) | 0.999 (0.995-1.002) | 0.375 | 0.998 (0.989-1.004) | 0.458 |
| γ-Linolenic acid (18:3 n-6) | 0.999 (0.995-1.004) | 0.799 | 0.996 (0.990-1.004) | 0.345 |
| Stearidonic acid (18:4 n-3) | 1.000 (0.964-1.038) | 0.993 | 0.973 (0.905-1.045) | 0.450 |
| Eicosadienoic acid (20:2 n-6) | 1.014 (0.995-1.033) | 0.143 | 1.004 (0.978-1.031) | 0.753 |
| Dihomo-γ-Linolenic acid (20:3 n-6) | 1.000 (0.998-1.002) | 0.981 | 0.997 (0.993-1.002) | 0.241 |
| Eicosatrienoic acid (20:3 n-9) | 1.027 (1.008-1.045) | 0.004 | 1.002 (0.963-1.042) | 0.927 |
| Arachidonic acid (20:4 n-6) | 1.000 (1.000-1.001) | 0.841 | 1.000 (0.999-1.001) | 0.643 |
| Eicosapentaenoic acid (20:5 n-3) | 0.999 (0.997-1.001) | 0.271 | 0.998 (0.994-1.002) | 0.247 |
| Docosatetraenoic acid (22:4 n-6) | 1.024 (1.011-1.036) | <0.001 | 1.014 (0.997-1.031) | 0.104 |
| Docosapentaenoic acid (22:5 n-3) | 1.001 (0.995-1.007) | 0.836 | 0.999 (0.990-1.008) | 0.856 |
| Docosapentaenoic acid (22:5 n-6) | 1.018 (1.005-1.032) | 0.006 | 1.006 (0.983-1.030) | 0.592 |
| Docosahexaenoic acid (22:6 n-3) | 0.998 (0.996-1.000) | 0.009 | 0.997 (0.994-1.000) | 0.033 |
| Saturated fatty acids (N=3900) | | | | |
| Capric acid (10:0) | 1.020 (0.999-1.042) | 0.057 | 1.028 (0.986-1.071) | 0.199 |
| Lauric acid (12:0) | 1.000 (0.994-1.005) | 0.957 | 1.002 (0.992-1.012) | 0.706 |
| Myristic acid (14:0) | 1.002 (1.000-1.003) | 0.111 | 1.001 (0.998-1.005) | 0.494 |
| Pentadecanoic acid (15:0) | 1.008 (0.994-1.022) | 0.246 | 1.004 (0.976-1.032) | 0.787 |
| Palmitic acid (16:0) | 1.000 (1.000-1.000) | 0.022 | 1.000 (1.000-1.000) | 0.639 |
| Margaric acid (17:0) | 1.006 (0.991-1.022) | 0.431 | 1.012 (0.982-1.044) | 0.433 |
| Stearic acid (18:0) | 1.000 (1.000-1.001) | 0.276 | 1.000 (0.998-1.001) | 0.775 |
| Arachidic acid (20:0) | 0.992 (0.972-1.013) | 0.463 | 0.983 (0.944-1.024) | 0.418 |
| Docosanoic acid (22:0) | 0.994 (0.987-1.000) | 0.060 | 0.993 (0.980-1.006) | 0.297 |
| Tricosanoic acid (23:0) | 0.975 (0.959-0.991) | 0.002 | 0.986 (0.955-1.017) | 0.369 |
| Lignoceric acid (24:0) | 0.992 (0.984-0.999) | 0.035 | 0.989 (0.973-1.004) | 0.152 |

Supplementary Table S5. Association of quartile concentrations of serum fatty acids with cardiovascular mortality in different gender population.

|  | All | | Male | | Female | |
| --- | --- | --- | --- | --- | --- | --- |
|  | HR (95%CI) | *P* | HR (95%CI) | *P* | HR (95%CI) | *P* |
| Docosahexaenoic acid (22:6 n-3) | | | | | | |
| Q1 (<109) | 1 (ref) |  | 1 (ref) |  | 1 (ref) |  |
| Q2 (109-143) | 0.820 (0.593-1.132) | 0.227 | 0.809 (0.346-1.894) | 0.626 | 2.318 (0.738-7.279) | 0.150 |
| Q3 (144-198) | 1.065 (0.790-1.437) | 0.679 | 1.264 (0.594-2.690) | 0.542 | 2.600 (0.848-7.976) | 0.095 |
| Q4 (≥199) | 0.970 (0.714-1.318) | 0.846 | 1.173 (0.525-2.618) | 0.697 | 1.396 (0.420-4.637) | 0.586 |

Abbreviations: Q1, below 25th percentile; Q2, between 25th percentile and 50th percentile; Q3, between 50th percentile and 75th percentile; Q4, above 75th percentile;

HR, hazard ratio; CI, confidence interval; ref, reference.

Supplementary Figure S1. Restricted Cubic Spline analysis for association of serum fatty acids with cardiovascular mortality.
